# "The contribution of chronic diseases to the prevalence of dependence among older people in Latin America, China and India: a 10/66 Dementia Research Group population-based survey"

**DOI:** 10.1186/1471-2318-10-53

**Published:** 2010-08-06

**Authors:** Renata M Sousa, Cleusa P Ferri, Daisy Acosta, Mariella Guerra, Yueqin Huang, KS Jacob, AT Jotheeswaran, Milagros A Guerra Hernandez, Zhaorui Liu, Guillermina Rodriguez Pichardo, Juan J Llibre Rodriguez, Aquiles Salas, Ana Luisa Sosa, Joseph Williams, Tirso Zuniga, Martin Prince

**Affiliations:** 1 King's College London, Institute of Psychiatry, Health Services and Population Research Department, Centre for Public Mental Health, De Crespigny Park, Po Box 60, SE5 8AF, London, UK; 2 Universidad Nacional Pedro Henriquez Ureña (UNPHU), Internal Medicine Department, Geriatric Section, Santo Domingo, Dominican Republic; 3 Psychogeriatric Unit, National Institute of Mental Health "Honorio Delgado Hideyo Noguchi", Lima, Peru; 4 Peking University, Institute of Mental Health, Beijing, China; 5 Christian Medical College, Vellore, India; 6 Institute of Community Health, Voluntary Health Services, Chennai, India; 7 Policlinico Universitario 27 de Noviembre, Marianao Ciudad Habana, Cuba; 8 Colegio Dominicano de Estadisticos y Demografos (CODE), Santo Domingo, Dominican Republic; 9 Facultad de Medicina Finlay-Albarran, Medical University of Havana, Havana, Cuba; 10 Medicine Department, Caracas University Hospital, Faculty of Medicine, Universidad Central de Venezuela, Caracas, Venezuela; 11 National Institute of Neurology and Neurosurgery of Mexico, National University Autonomous of Mexico, Mexico City, Mexico

## Abstract

**Background:**

The number of older people is set to increase dramatically worldwide. Demographic changes are likely to result in the rise of age-related chronic diseases which largely contribute to years lived with a disability and future dependence. However dependence is much less studied although intrinsically linked to disability. We investigated the prevalence and correlates of dependence among older people from middle income countries.

**Methods:**

A one-phase cross-sectional survey was carried out at 11 sites in seven countries (urban sites in Cuba, Venezuela, and Dominican Republic, urban and rural sites in Peru, Mexico, China and India). All those aged 65 years and over living in geographically defined catchment areas were eligible. In all, 15,022 interviews were completed with an informant interview for each participant. The full 10/66 Dementia Research Group survey protocol was applied, including ascertainment of depression, dementia, physical impairments and self-reported diagnoses. Dependence was interviewer-rated based on a key informant's responses to a set of open-ended questions on the participant's needs for care. We estimated the prevalence of dependence and the independent contribution of underlying health conditions. Site-specific prevalence ratios were meta-analysed, and population attributable prevalence fractions (PAPF) calculated.

**Results:**

The prevalence of dependence increased with age at all sites, with a tendency for the prevalence to be lower in men than in women. Age-standardised prevalence was lower in all sites than in the USA. Other than in rural China, dementia made the largest independent contribution to dependence, with a median PAPF of 34% (range 23%-59%). Other substantial contributors were limb impairment (9%, 1%-46%), stroke (8%, 2%-17%), and depression (8%, 1%-27%).

**Conclusion:**

The demographic and health transitions will lead to large and rapid increases in the numbers of dependent older people particularly in middle income countries (MIC). The prevention and control of chronic neurological and neuropsychiatric diseases and the development of long-term care policies and plans should be urgent priorities.

## Background

The number of people worldwide aged 60 years and over will reach two billion by 2050 [1]. Most will be living in low or middle income countries (LMIC), where chronic diseases are already responsible for the majority of the total disease burden [2]. Chronic diseases tend to be age-dependent, and are particularly characterised by their contribution to years lived with disability [3,4]. As the epidemiological and demographic transitions progress, health systems in LMIC will need increasingly to prioritise the prevention and control of these conditions, and manage their long-term consequences. Disability has been widely studied, particularly through the Global Burden of Disease Report; dependence, defined as 'the need for frequent human help or care beyond that habitually required by a healthy adult' [5], much less so. They are related phenomena, in that disability is the root cause of dependence; however, not all those with disability have needs for care. A recent report from the World Health Organisation (WHO) Global Burden of Disease project on the global prevalence of dependence acknowledged the relative lack of empirical data, particularly from LMIC [5]. Both disability levels and needs for care were inferred ultimately from diagnoses; the total population prevalence of dependence was estimated to be similar worldwide, varying from 4.4% to 5.1% by region, and was predicted to increase only marginally by 2050 [5]. Direct estimates from population-based surveys are limited mainly to older people in high income countries (HIC), with a prevalence ranging between 15% and 17% of those aged 65 and over [6].

Dependence is an important, yet neglected topic in public health because of the significant consequences for the dependent person, their caregivers and wider society. The worldwide societal cost of dementia (a leading cause of dependence) was recently estimated as US$315 billion per year [7]. Informal care accounted for 56% of costs in low income countries, 42% in middle income countries, and 31% in high income countries [8]. In the USA, there are an estimated 44 million adult caregivers, two-thirds of whom care for a person aged 65 years or older [9]. The national economic value of informal caregiving was calculated as $196 billion in 1997, exceeding the combined national spending for formal home care and nursing home care [10]. Family and friends who provide care typically take pride in their role, and perceive many positives [11]. Nevertheless, according to various estimates, between 40% and 75% of carers of people with dementia have significant psychological morbidity [12,13], and 15% to 32% meet diagnostic criteria for major depression [14]. In LMIC [15] levels of carer strain are as high as those in Europe [12] despite extended family care networks. However, the reliability and universality of the family care system in LMIC is overstated [16,17]. Declining fertility rates, migration, the education of women and their increasing workforce participation limit the available pool of caregivers, and their willingness to take on this additional role. Social anthropologists have identified 'dependence anxiety' arising from the lack of a family to provide care in the event of deteriorating health, or fear of becoming a burden coupled with an expectation of inadequate support [18,19]. Under these circumstances, recourse to charity, homelessness or admission to the public hostel for the indigent may be the only available options [20].

An understanding of the determinants of dependence is an essential prerequisite for prevention, long-term care policymaking and planning. Older people are likely to have multiple health conditions - chronic physical diseases affecting different organ systems, coexisting with mental and cognitive disorders - interacting in complex ways to create difficulties in performing important tasks and activities, and in determining needs for care. Our previous analyses of data from the 10/66 Dementia Research Group studies in Cuba [21] and the Dominican Republic [6] indicated that while dependence was characterised by cognitive, physical and mental comorbidity, dementia made by far the largest independent contribution. In Nigeria [22] dementia was not studied; the effect of cognitive impairment was somewhat less, and the effect of depression somewhat more prominent.

The 10/66 Dementia Research Group has now completed comprehensive population-based cross-sectional surveys of catchment areas in Latin America, India, China and Africa [23]. The objectives of the present study were 1) to estimate the prevalence of dependence in middle income countries (MIC), comparing them with USA estimates, 2) to analyse the social patterning of dependence in MIC (age, sex and socio-economic status) and 3) to analyse the relative contribution of different chronic diseases to dependence in representative samples of the general older population in these settings.

## Methods

### The 10/66 Dementia Research Group population-based studies

One-phase population-based surveys were carried out, between 2003 and 2005, of all older people aged 65 years and over living in geographically defined catchment areas from seven developing countries (urban sites in Cuba, Dominican Republic and Venezuela, and urban and rural sites in Mexico, Peru, China and India) [23]. For urban catchment areas, predominantly middle-class or professional areas with high-income earners were avoided. Rural areas were defined by low population density and traditional agrarian lifestyle [23]. The 10/66 protocol for the baseline survey includes a clinical interview, an informant interview, and a physical examination. It generates information regarding dementia diagnosis, mental disorders, physical health, anthropometry, demographics, an extensive dementia and chronic diseases risk factor questionnaire, disability, health service utilisation, care arrangements and caregiver strain. Only the assessments relevant to the current analyses of the prevalence and correlates of dependence will be described in detail here. The sample size for each country was between 2000 and 3000. All studies were approved by local ethical committees and by the King's College London research ethics committee.

### Measures

1. Dependence. The interviewer administered open-ended questions to a key informant, to ascertain dependence: Who shares the home with the participant? What kind of help does the participant need inside and outside of the home? Who, in the family, is available to care for the participant? What help do you provide? Do you help to organise care for the participant? Is there anyone else in the family who is more involved in helping than you? What do they do? What about friends and neighbours? What do they do? The interviewer then coded whether the participant required no care, care some of the time, or care much of the time. This coding was based upon the interviewer's perception of needs for care, independent of whether these were routinely met. Key informants were selected by interviewers on the basis of who knew the old person best, and could give the clearest and most detailed account of their current circumstances. The priority were co-residents and family members unless others were clearly better qualified. The main criterion for selection in case of several co-resident family members was time spent with the older person. In cases where the older person needed care, then the main caregiver was selected. However, if the main caregiver was paid, the main organisational caregiver was selected instead.

2. Socio-demographic characteristics. Information on age, sex, marital status, level of education (none; some, but did not complete primary; completed primary; completed secondary; completed tertiary or further education) and living circumstances (living with children, yes/no) was assessed by a standard socio-demographic questionnaire.

3. Directly assessed diagnoses. a) Dementia was ascertained according to the cross-culturally validated 10/66 dementia diagnosis algorithm [24] and the DSM-IV dementia criterion [25]. b) Depression according to ICD-10 criteria (depressive episode; mild, moderate or severe) ascertained using the Geriatric Mental State examination (GMS) [26]. c) Hypertension according to the European Society of Hypertension criteria (systolic blood pressure >=140 mm Hg and/or diastolic blood pressure >=95 mm Hg, and/or a positive answer to the question "have you ever been told by a doctor that you have hypertension?". d) Chronic Obstructive Pulmonary Disease (COPD) defined as having chronic cough, productive of sputum for three or more months.

4. Self-reported diagnoses. The ascertainment of previous episodes of stroke or ischaemic heart disease was based on self-report ("have you ever been told by a doctor that you had a stroke/angina/heart attack?")

5. Physical impairments. Self-reported paralysis, weakness or loss of a limb; eyesight problems; stomach or intestine problems; arthritis or rheumatism; heart problems; hearing difficulties or deafness; breathlessness; difficulty breathing or asthma; faint or blackouts; skin disorders such as pressure sores, leg ulcers or severe burns; persistent cough. Impairments were rated as present if they interfered with activities 'a little' or 'a lot' [27].

### Statistical Analyses

We used the 10/66 data archive (release 2.0; February 2009) and STATA (version 10.0) [28] for all analyses.

1) We report the prevalence of dependence (needing some or much care) by age and sex, generating robust standard errors and 95% confidence intervals accounting for household clustering. We used standardisation to compare: a) the prevalence of dependence among the 10/66 sites having adjusted for the compositional effects of age, sex, education and chronic disease (direct standardization, with the whole sample as the standard population); b) the prevalence of dependence in each of the 10/66 sites, with that from USA National Long Term Care Survey [29] (indirect standardization for age-standardized morbidity ratios (SMR) for dependence and Fieller 95% confidence intervals were calculated with an SMR of 100 for the reference population).

2) We modelled the effects of age, sex and education, providing mutually adjusted prevalence ratios derived from a Poisson working model. We fitted the model separately for each site and then used a fixed effects meta-analysis to combine them, estimating the degree of heterogeneity using Higgins' I^2 ^[30] with approximate 95% confidence intervals. I^2 ^values smaller than 30% signify mild heterogeneity whereas values exceeding 56% imply severe heterogeneity [30].

3) We generated Poisson regression working models to estimate the independent contributions of health conditions (self-reported impairments and diagnoses) to dependence for each site, controlling for age, sex, education and marital status and all health conditions, adjusted for household clustering. We then calculated a population attributable prevalence fraction (PAPF) for the association between dependence and each of the health conditions using the STATA aflogit command which estimates the attributable fraction from within the Poisson regression framework, thus enabling confounders to be taken into account. Population attributable prevalence fractions when calculated from prevalence ratios in cross-sectional studies represent the proportion of prevalent severe dependence that could theoretically be avoided if the exposure could be removed from the population, taking into account the effect of the exposure on both incidence and duration of the severe dependent state, assuming a causal relationship estimated free of confounding. Finally, we again used fixed-effect meta-analysis to pool the associations between dependence and health conditions across sites.

## Results

In all, 15,022 interviews were completed at the 11 sites in seven countries. Response proportions (Table 1) varied between 72% and 98%, and were 80% or higher in all but two sites (urban China and urban India).

**Table 1:** Participants Socio-Demographic and Health Characteristics

*Variable*	*Cuba *	*Dominican Republic*	*Urban Peru*	*Rural Peru*	*Venezuela *	*Urban Mexico*	*Rural Mexico*	*Urban China*	*Rural China*	*India Urban *	*Rural India*
**Response rate (%)**	94	95	80	88	80	84	86	74	96	72	98
**Achieved sample (n)**	2,944	2,011	1,381	552	1,965	1,003	1,000	1,160	1,002	1,005	999
**Age (MV)**	7	0	1	0	4	1	0	0	0	4	0
Mean (SD)	75.1 (7.0)	75.3 (7.5)	75.0 (7.4)	74.2 (7.3)	72.3 (6.9)	74.5 (6.6)	74.1 (6.7)	73.9 (6.2)	72.4 (6.0)	71.3 (6.1)	72.6 (5.8)
**Sex (MV)**	0	2	0	0	33	0	0	0	0	15	0
Female (%)	1913 (65.0)	1325 (65.9)	888 (64.3)	295 (53.4)	1226 (63.5)	666 (66.4)	602 (60.2)	661 (57.0)	556 (55.5)	571 (57.7)	545 (54.6)
**Education (MV)**	8	19	8	8	40	2	0	0	0	2	0
No education (%)	75 (2.6)	392 (19.7)	37 (2.7)	84 (15.4)	156 (8.1)	227 (22.7)	327 (32.7)	232 (20.0)	579 (57.8)	428 (42.7)	660 (66.1)
Some Education (%)	655 (22.3)	1022 (51.3)	90 (6.6)	141 (25.9)	445 (23.1)	354 (35.4)	510 (51.0)	153 (13.2)	114 (11.4)	234 (23.3)	195 (19.5)
Primary (%)	979 (33.3)	370 (18.6)	460 (33.5)	267 (49.1)	965 (50.1)	229 (22.9)	122 (12.2)	303 (26.1)	259 (25.9)	212 (21.1)	116 (11.6)
Secondary (%)	728 (24.8)	135 (6.8)	481 (35.0)	36 (6.6)	266 (13.8)	99 (9.9)	25 (2.5)	335 (28.9)	45 (4.5)	87 (8.7)	26 (2.6)
Tertiary (%)	499 (17.0)	73 (3.7)	305 (22.2)	16 (2.9)	93 (4.8)	92 (9.2)	16 (1.6)	137 (11.8)	5 (0.5)	42 (4.2)	2 (0.2)
**Currently married (MV)**	8	15	10	1	45	0	1	0	0	3	0
Currently married	1271 (43.3)	586 (29.4)	784 (57.2)	308 (55.9)	921 (48.0)	470 (46.9)	538 (53.9)	829 (71.5)	585 (58.4)	523 (52.2)	481 (48.1)
**Co residence (MV)**	0	0	0	0	0	0	0	0	0	0	0
With children (%)	1422 (48.3)	963 (47.9)	890 (64.4)	326 (59.1)	1578 (80.3)	565 (56.3)	523 (52.3)	446 (38.4)	679 (67.8)	719 (71.5)	625 (62.6)
**Dementia (MV)**	0	0	0	0	0	0	0	0	0	0	0
10/66 or DSM-IV criteria (%)	323 (11.0)	242 (12.0)	130 (9.4)	36 (6.5)	145 (7.4)	93 (9.3)	87 (8.7)	84 (7.2)	56 (5.6)	75 (7.5)	108 (10.8)
**Depression (MV)**	0	0	0	0	0	0	0	0	0	0	0
ICD-10 depressive episode (%)	144 (4.9)	278 (13.8)	87 (6.3)	16 (2.9)	107 (5.5)	47 (4.7)	45 (4.5)	3 (0.3)	7 (0.7)	39 (3.9)	126 (12.6)
**Physical impairments (MV)**	6	2	1	1	33	0	0	0	0	1	0
Three or more (%)	292 (9.9)	465 (23.1)	224 (16.2)	40 (7.3)	489 (24.9)	158 (15.8)	185 (18.5)	208 (17.9)	39 (3.9)	41 (4.1)	168 (16.8)
**Self-reported health (MV)**	9	2	7	1	46	1	0	0	0	2	0
'bad' or 'very bad' (%)	271 (9)	186 (9)	70 (5)	11 (2)	87 (5)	87 (9)	84 (8)	42 (4)	42 (4)	46 (5)	97 (10)
**Informant co-resident (MV)**	24	3	1	3	15	0	0	0	0	4	0
Yes (%)	2307 (78.4)	1388 (69.1)	1166 (84.4)	460 (83.3)	1633 (83.1)	778 (77.6)	619 (61.9)	1064 (91.7)	960 (95.8)	807 (80.3)	806 (80.7)
**Informant age group (MV)**	21	5	1	6	11	2	2	0	0	4	0
≤20 years (%)	68 (2.3)	107 (5.3)	25 (1.8)	13 (2.4)	62 (3.2)	32 (3.2)	50 (5.0)	13 (1.2)	3 (0.3)	39 (3.8)	26 (2.6)
21-44 years (%)	941 (31.9)	731 (36.3)	340 (24.6)	216 (39.2)	748 (38.1)	393 (39.2)	551 (55.1)	177 (15.3)	275 (27.5)	500 (49.7)	492 (49.2)
45-64 years (%)	912 (30.9)	674 (33.5)	421 (30.5)	161 (29.2)	711 (36.2)	321 (32.0)	315 (31.5)	270 (23.3)	399 (39.8)	315 (31.4)	361 (36.2)
≥65 years (%)	1002 (34.1)	494 (24.6)	594 (43.1)	156 (28.3)	433 (22.1)	255 (25.4)	82 (8.2)	700 (60.4)	325 (32.4)	147 (14.6)	120 (12.1)
**Informant Sex (MV)**	26	3	0	3	22	1	0	0	0	5	0
Female (%)	2033 (69.1)	1454 (72.3)	996 (72.2)	398 (72.1)	1366 (69.5)	740 (73.8)	799 (79.9)	666 (57.4)	341 (34.1)	758 (75.4)	826 (82.7)
**Informant relationship to** **older participant (MV)**	21	5	1	3	22	1	0	1	0	5	0
Spouse (%)	926 (31.4)	403 (20.0)	463 (33.5)	162 (29.3)	530 (26.9)	224 (22.3)	104 (10.4)	691 (59.6)	345 (34.4)	264 (26.3)	328 (32.8)
Child (%)	1057 (35.9)	738 (36.7)	505 (36.6)	257 (46.6)	928 (47.2)	497 (49.5)	480 (48.0)	317 (27.3)	521 (52.0)	297 (29.5)	200 (20.0)
Son-Daughter-in Law (%)	99 (3.4)	50 (2.5)	32 (2.3)	19 (3.4)	61 (3.1)	70 (6.9)	177 (17.7)	42 (3.6)	108 (10.8)	253 (25.2)	297 (29.7)
Sibling (%)	145 (4.9)	108 (5.4)	98 (7.1)	23 (4.2)	54 (2.7)	40 (3.9)	32 (3.2)	5 (0.4)	3 (0.3)	24 (2.4)	15 (1.5)
Other (%)	696 (23.6)	707 (35.2)	282 (20.4)	88 (15.9)	370 (18.8)	171 (17.0)	207 (20.7)	104 (8.9)	25 (2.5)	162 (16.2)	159 (15.9)

Abbreviations used: MV = missing values; ICD-10 = International Classification of Diseases (10^th ^ed.)

### Sample characteristics

The mean ages of the samples varied between 71.3 and 75.1 years, demographic ageing being more advanced in the Latin American centres and in urban China, compared with rural China and India. Women predominated over men in all sites accounting for between 53% and 66% of the sample. Educational levels were highest in the urban sites in Cuba (3% having no education), Peru (3%) and Venezuela (8%). In the Dominican Republic, rural Peru and rural and urban Mexico a fifth to a third lacked any education, whilst in rural China (58%) and rural (66%) and urban India (43%) having no education was the norm. The most prevalent self-reported physical impairments were eyesight problems (median prevalence 28.4%, range 6.5% to 39.6%), arthritis/rheumatism (18.2%, 1.9% to 51.1%), hearing difficulties (14.2%, 3.1% to 22.9%), and stomach/intestine problems (8.7%, 1.2% to 19.3%). Paralysis/weakness of limb, heart problems, difficulty breathing/asthma, faint or blackouts, skin disorders and persistent cough were reported by fewer than 10% of participants in almost all sites. The most common diagnosis was hypertension (median prevalence 62.6%, range 28.5% to 75.4%), followed by dementia (8.7%, 5.6% to 12.0%), stroke (7.1%, 1.1% to 8.7%), COPD (5.8%, 1.6% to 7.6%), depression (4.7%, 0.3% to 13.8%) and ischaemic heart disease (4.4%, 1.2% to 14.2%).

Table 1 also shows that in all sites most informants were coresident with the older participant (range between 61.9% in rural Mexico and 95.8% in rural China). With the exception of rural China (34.1%) the large majority were female. In most sites between a fifth to a third of informants were the spouses of the older participants. In all sites other than urban China most informants were the children or children-in-law of the older participant.

### Prevalence of dependence by age and sex and the effect of education

The crude prevalence of dependence varied from 2.9% in urban India to 15.7% in urban China (Table 2), and, with the exception of India, was lower in rural than urban catchment areas. The prevalence of dependence increased with age in all sites (meta-analysed PR 1.83, 95% CI 1.74-1.93). There was a tendency for the prevalence to be lower in men than in women (0.83, 0.75-0.95), particularly in older age groups. Those with better education tended to have a lower prevalence of dependence (0.89, 0.84-0.94), although the trend was in the opposite direction in rural Mexico.

**Table 2:** Prevalence of dependence (needs for care) in percentage by age and sex across the 10/66 DRG sites and standardized prevalence*

		*65-69*	*70-74*	*75-79*	*80+*	*Age*	*Sex*	*Education*	*Crude* *Prevalence* *(95% CI)*	*Standardized* *Morbidity Ratio* *(95% CI) *†
						Adjusted*	Adjusted*	Adjusted*		

**Cuba**	Female	3.1% (1.4-4.6)	8.1% (5.5-10.7)	6.6% (3.9-9.2)	26.9% (22.8-31.1)	2.04 (1.76-2.34)	0.68 (0.53-0.89)	0.84 (0.73-0.95)	10.0% (8.9-11.2)	55.5 (49.1-62.6)
	Male	2.8% (0.76-4.9)	2.2% (0.46-4.0)	5.4% (2.3-8.4)	20.4% (14.9-25.7)					
**Dominican Republic**	Female	2.6% (0.9-4.3)	8.4% (5.3-11.4)	7.9% (4.6-11.2)	29.6% (25.1-34.1)	1.88 (1.65-2.14)	0.79 (0.61-1.05)	0.92 (0.78-1.06)	11.8% (10.4-13.2)	62.9 (55.3-71.4)
	Male	4.3% (1.4-7.2)	7.2% (3.5-10.8)	12.1% (6.5-17.7)	15.5% (9.9-20.9)					
**Urban Peru**	Female	2.2% (0.5-4.1)	5% (2.1-7.9)	7.4% (3.6-11.1)	26.6% (20.7-32.6)	2.06 (1.72-2.47)	0.89 (0.63-1.26)	0.92 (0.77-1.11)	9.7% (8.1-11.4)	53.5 (45.2-63.3)
	Male	5.4% (1.1-9.5)	4.5% (0.96-8.1)	8.3% (3.1-13.6)	17.7% (11.4-24.1)					
**Rural Peru**	Female	1% (0-2.9)	2.4% (0-5.7)	7.5% (0.3-14.7)	12.1% (3.5-20.5)	1.58 (1.12-2.24)	0.89 (0.37-2.11)	0.82 (0.53-1.26)	4.7% (2.9-6.5)	27.5 (18.8-40.5)
	Male	2.5% (0-6)	5.2% (0-10.9)	6.4% (0-13.5)	5.5% (0.2-10.9)					
**Venezuela**	Female	3.3% (1.7-4.8)	5.5% (2.9-8.2)	13.8% (9.3-18.5)	31.8% (25.4-38.3)	1.96 (1.72-2.25)	0.77 (0.57-1.05)	0.87 (0.74-1.02)	10.6% (9.2-12.0)	71.5 (62.5-81.9)
	Male	3.5% (1.4-5.5)	5.6% (2.2-9.1)	11.3% (5.7-16.9)	17.2% (9.2-25.2)					
**Urban Mexico**	Female	3.7% (1.0-6.5)	7.1% (3.6-10.6)	14.3% (8.2-20.4)	27.9% (20.6-35.4)	1.71 (1.41-2.06)	0.79 (0.55-1.15)	0.74 (0.62-0.88)	11.3% (9.2-13.5)	67.5 (56.2-81.1)
	Male	5% (0-10.6)	8.5% (3.4-13.5)	7.6% (1.7-13.5)	17.5% (9.1-25.9)					
**Rural Mexico**	Female	6.1% (2.7-9.4)	6.7% (2.7-10.7)	8.2% (3.5-12.8)	14.7% (8.4-21.1)	1.43 (1.17-1.73)	0.85 (0.56-1.26)	1.21 (0.95-1.54)	8.2% (6.4-9.9)	48.7 (39.2-60.5)
	Male	2.9% (0-6.3)	6.8% (1.9-11.7)	9.2% (3.1-15.3)	12.3% (5.9-18.5)					
**Urban China**	Female	5.4% (2.3-8.5)	11.4% (7.0-15.8)	18.9% (12.4-25.6)	42.0% (33.1-50.9)	1.85 (1.62-2.12)	0.84 (0.64-1.11)	0.93 (0.83-1.05)	15.7% (13.6-17.9)	98.3 (85.1-113.7)
	Male	8.9% (3.6-14.3)	6.2% (2.5-9.9)	17.9% (10.9-24.9)	29.4% (20.7-37.9)					
**Rural China**	Female	1.1% (0-2.5)	4.8% (1.5-8.2)	4.8% (1.1-8.6)	14.6% (6.6-22.7)	1.86 (1.49-2.33)	1.48 (0.89-2.46)	0.94 (0.72-1.23)	5.4% (3.9-6.8)	38.2 (28.7-49.9)
	Male	2.1% (0.05-4.1)	5.3% (1.4-9.2)	15.6% (7.4-23.7)	8.7% (0.4-16.9)					
**Urban India**	Female	1.3% (0-2.7)	3.2% (0.7-5.7)	1.4% (0-4.1)	7.7% (1.2-14.2)	1.94 (1.42-2.64)	1.21 (0.62-2.37)	1.01 (0.73-1.39)	2.9% (1.8-4.01)	21.5 (14.4-30.9)
	Male	0.6% (0-1.7)	2.4% (0-5.2)	7.4% (1.1-13.8)	10% (1.6-18.4)					
**Rural India**	Female	6.7% (3.2-10.3)	8.2% (4.3-12.1)	8.9% (3.0-14.9)	27.9% (17.2-38.7)	1.43 (1.17-1.75)	0.98 (0.63-1.53)	0.43 (0.25-0.72)	8.5% (6.7-10.2)	60.3 (48.2-74.6)
	Male	5.1% (1.4-8.7)	6.5% (2.6-10.4)	3.4% (0-7.2)	12.3% (4.7-19.9)					

* Adjusted for age, sex and education; † Standardized for age, using US National Long-Term Care Study estimates as comparison. 95% CIs calculated using Boice-Monson Method based on Rothman and Greenland, Modern Epidemiology, 2^nd ^edition, 1998

Age-standardized morbidity ratios (SMR) for dependence showed that the prevalence of dependence in our MIC sites was generally between one half to three-quarters of that in the USA. The SMRs for urban India (21.5), rural Peru (27.5) and rural China (38.2) were strikingly low, while that for urban China (98.3) indicated a similar prevalence to the USA reference population.

### Associations between dependence and health conditions

Table 3 shows the independent associations between health conditions and dependence. In order of strength of association (judged by population attributable prevalence fraction) only dementia, paralysis or weakness of limbs, stroke, depression, eyesight problems and arthritis or rheumatism were significantly associated with dependence. Dependence was independently 2.8 to 9.5 times more common among those with dementia (meta-analysed PR 4.49, 95% CI 3.98-5.07). In every site, other than rural China, dementia made the largest contribution to dependence with a median PAPF of 34% and a range of 23% to 59%. The next most substantial contribution to dependence was from paralysis/weakness of limb (median PAPF 9%, range 1% to 46%). While significant heterogeneity was observed in the effects of dementia and limb paralysis/weakness on dependence, this was only in the size of the positive association, which, in the case of dementia, varied from substantial to very substantial. Ischaemic heart disease, hypertension, respiratory disease, gastrointestinal problems, deafness and skin diseases were not independently associated with dependence. Chronic disease diagnoses and impairments accounted collectively for a PAPF of between 40.2% and 74.2% by site.

**Table 3:** Prevalence ratios^¶ ^for the associations between dependence and self-reported impairments and diagnoses, by site

	*Cuba*	*Dominican Republic*	*Urban Peru*	*Rural Peru*	*Venezuela*	*Urban Mexico*	*Rural Mexico*	*Urban China*	*Rural China*	*Urban India*	*Rural India*	*Meta-analysed PR (95% CI)*	*Cochran's Q Higgins I^2 ^(95% CI)*	*PAPF median (range)*
**Dementia**	9.46 (7.01-12.7)	2.79 (2.11-3.68)	6.76 (4.22-10.83)	8.05 (2.93-22.1)	4.21 (3.05-5.82)	4.55 (3.06-6.75)	3.58 (2.28-5.64)	3.60 (2.60-4.9)	5.35 (3.09-9.29)	9.29 (3.72-23.2)	2.87 (1.83-4.51)	4.49 (3.98-5.07)	Q = 48.69 I^2 ^= 79%	34% (23-59)
**PAPF**	59%	27%	47%	38%	28%	34%	23%	29%	42%	45%	24%		(64-88)	
**Paralysis/weakness**	1.87 (1.36-2.57)	3.77 (2.82-5.03)	2.02 (1.08-3.77)	4.94 (1.63-14.9)	1.13 (0.69-1.83)	1.95 (1.13-3.37)	3.40 (2.04-5.67)	3.01 (1.89-4.79)	13.27 (7.24-24.3)	1.76 (0.22-13.9)	2.90 (1.33-6.30)	2.76 (2.38-3.20)	Q = 53.42 I^2 ^= 81%	9% (1-46)
**PAPF**	6%	20%	9%	6%	2%	5%	10%	20%	46%	1%	12%		(68-89)	
**Stroke**	1.78 (1.36-2.33)	1.27 (0.9-1.76)	2.40 (1.43-4.03)	3.01 (0.69-13.0)	2.04 (1.41-2.95)	1.92 (1.19-3.11)	2.60 (1.62-4.17)	1.31 (0.84-2.1)	1.23 (0.59-2.57)	3.86 (1.40-10.6)	2.87 (1.13-7.33)	1.78 (1.55-2.05)	Q = 15.10 I^2 ^= 34%	8% (2-17)
**PAPF**	10%	5%	17%	7%	11%	8%	13%	7%	2%	10%	6%		(0-67)	
**Depression**	1.56 (1.08-2.24)	2.79 (2.11-3.68)	2.06 (1.35-3.15)	5.25 (1.51-18.2)	2.30 (1.43-3.69)	0.93 (0.47-1.83)	2.55 (1.52-4.27)	1.00 (0.41-2.43)	1.11 (0.61-2.02)	2.57 (0.89-7.42)	1.84 (1.03-3.29)	1.70 (1.46-1.98)	Q = 15.63 I^2 ^= 36%	8% (0-27)
**PAPF**	3%	27%	7%	16%	8%	0	8%	0	1%	10%	10%		(0-69)	
**Eyesight problems**	1.21 (0.97-1.50)	1.40 (1.12-1.84)	1.20 (0.82-1.75)	0.72 (0.30-1.74)	0.95 (0.70-1.29)	1.36 (0.96-1.94)	0.92 (0.59-1.45)	1.18 (0.85-1.64)	1.79 (0.94-3.39)	2.06 (0.46-9.13)	1.12 (0.70-1.80)	1.20 (1.08-1.34)	Q = 9.37 I^2 ^= 0 (0-60)	6% (0-16)
**PAPF**	7%	16%	7%	0	0	11%	0	3%	6%	7%	3%			
**Arthritis/rheumatism**	1.18 (0.93-1.50)	0.93 (0.72-1.20)	1.29 (0.89-1.88)	0.53 (0.11-2.50)	1.87 (0.85-1.65)	1.32 (0.85-2.04)	0.87 (0.56-1.37)	1.50 (1.07-2.10)	0.71 (0.15-3.28)	1.05 (0.42-2.59)	1.07 (0.66-1.74)	1.13 (1.01-1.27)	Q = 8.73 I^2 ^= 0 (0-60)	4% (0-6)
**PAPF**	4%	0	5%	0	5%	5%	0	6%	0	0	4%			
***Stomach/intestine problems***	*0.86* *(0.62-1.18)*	*1.10* *(0.82-1.48)*	*1.01* *(0.67-1.54)*	*3.71* *(1.13-12.2)*	*0.72* *(0.50-1.04)*	*1.10* *(0.69-1.76)*	*1.98* *(1.29-3.05)*	*1.12* *(0.72-1.74)*	*4.22* *(1.08-16.4)*	*NC*	*1.39* *(0.74-2.59)*	*1.09* *(0.95-1.25)*	*Q = 23.16* *I^2 ^= 61%* *(22-81)*	*1.5%* *(0-16)*
***PAPF***	*0*	*2%*	*0*	*11%*	*0*	*1%*	*16%*	*0*	*4%*		*2%*			
***Hearing difficulty***	*1.07* *(0.82-1.39)*	*1.32* *(0.99-1.75)*	*0.95* *(0.65-1.38)*	*1.31* *(0.40-4.28)*	*1.10* *(0.75-1.59)*	*0.98* *(0.66-1.46)*	*0.83* *(0.52-1.34)*	*1.04* *(0.75-1.46)*	*0.97* *(0.52-1.80)*	*NC*	*1.09* *(0.66-1.80)*	*1.06* *(0.92-1.21)*	*Q = 4.38* *I^2 ^= 0 (0-62)*	*1%* *(0-5)*
***PAPF***	*1%*	*5%*	*0*	*4%*	*2%*	*0*	*0*	*1%*	*0*		*2%*			
***Difficulty breathing***	*1.17* *(0.73-1.89*	*0.91* *(0.63-1.33)*	*1.39* *(0.72-2.68)*	*0.46* *(0.12-1.65)*	*1.22* *(0.78-1.91)*	*1.47* *(0.70-3.11)*	*1.04* *(0.46-2.34)*	*1.51* *(0.93-2.46)*	*1.45* *(0.39-5.36)*	*0.75* *(0.12-4.43)*	*1.47* *(0.83-2.60)*	*1.18* *(0.98-1.41)*	*Q = 6.42* *I^2 ^= 0 (0-60)*	*1%* *(0-6)*
***PAPF***	*0*	*0*	*2%*	*0*	*2%*	*2%*	*0*	*2%*	*1%*	*0*	*6%*			
***Heart problems ***	*0.97* *(0.66-1.43)*	*1.34* *(0.88-2.04)*	*1.09* *(0.60-1.99)*	*2.29* *(0.44-11.7)*	*0.76* *(0.45-1.27)*	*1.03* *(0.41-2.57)*	*1.01* *(0.28-3.59)*	*1.41* *(1.04-1.91)*	*1.54* *(0.56-4.18)*	*NC*	*NC*	*1.17* *(0.98-1.39)*	*Q = 6.51* *I^2 ^= 0 (0-65)*	*0* *(0-9)*
***PAPF***	*0*	*2%*	*0*	*4%*	*0*	*0*	*0*	*9%*	*1%*					
***Faint/blackout***	*1.13* *(0.69-1.83)*	*0.90* *(0.53-1.53)*	*1.09* *(0.45-2.59)*	*0.12* *(0.01-1.31)*	*1.46* *(0.86-2.47)*	*1.95* *(0.35-10.6)*	*0.89* *(0.33-2.37)*	*1.04* *(0.61-1.77)*	*0.51* *(0.19-1.38)*	*0.46* *(0.03-7.24)*	*1.78* *(1.04-3.05)*	*1.11* *(0.90-1.37)*	*Q = 12.01* *I^2 ^= 17% *	*0* *(0-11)*
***PAPF***	*0*	*0*	*0*	*0*	*3%*	*0*	*0*	*0*	*0*	*0*	*11%*		*(0-57)*	
***COPD†***	*0.88* *(0.51-1.50)*	*0.86* *(0.55-1.33)*	*0.58* *(0.32-1.06)*	*1.10* *(0.35-3.47)*	*1.14* *(0.75-1.73)*	*0.58* *(0.27-1.27)*	*1.49* *(0.76-2.90)*	*2.11* *(1.42-3.2)*	*1.04* *(0.46-2.37)*	*NC*	*0.86* *(0.41-1.81)*	*1.08* *(0.91-1.30)*	*Q = 20.63* *I^2 ^= 56% *	*0* *(0-4)*
***PAPF***	*0*	*0*	*0*	*0*	*1%*	*0*	*4%*	*4%*	*0*		*0*		*(12-78)*	
***Skin disorders***	*0.84* *(0.51-1.40)*	*2.57* *(1.63-4.03)*	*1.11* *(0.69-1.81)*	*0.26* *(0.06-1.05)*	*0.99* *(0.64-1.53)*	*0.47* *(0.14-1.58)*	*1.10* *(0.60-2.00)*	*0.38* *(0.18-0.79)*	*NC*	*NC*	*0.84* *(0.33-2.14)*	*1.05* *(0.86-1.29)*	*Q = 28.73* *I^2 ^= 72% *	*0* *(0-4)*
***PAPF***	*0*	*4%*	*1%*	*0*	*0*	*0*	*0*	*0*			*0*		*(45-86)*	
***Infarction/Angina‡***	*1.01* *(0.77-1.33)*	*1.80* *(1.13-2.88)*	*0.80* *(0.43-1.50)*	*1.09* *(0.26-4.40)*	*1.26* *(0.72-2.21)*	*1.37* *(0.53-3.54)*	*0.58* *(0.07-4.31)*	*0.66* *(0.43-1.1)*	*NC*	*0.87* *(0.10-7.08)*	*0.46* *(0.09-2.25)*	*1.02* *(0.85-1.21)*	*Q = 12.41* *I^2 ^= 28% *	*0* *(0-3)*
***PAPF***	*0*	*3%*	*0*	*0*	*2%*	*1%*	*0*	*0*		*0*	*0*		*(0-65)*	
***Persistent cough***	*0.85* *(0.40-1.76)*	*0.89* *(0.62-1.28)*	*1.04* *(0.63-1.73)*	*2.30* *(0.72-7.32)*	*0.89* *(0.56-1.39)*	*0.94* *(0.51-1.72)*	*0.52* *(0.17-1.54)*	*0.77* *(0.47-1.25)*	*NC*	*NC*	*1.06* *(0.52-2.12)*	*0.94* *(0.79-1.13)*	*Q = 5.24* *I^2 ^= 0 (0-65)*	*0* *(0-2)*
***PAPF***	*0*	*0*	*0*	*2%*	*0*	*0*	*0*	*0*			*0*			
***Hypertension***	*0.79* *(0.64-0.99)*	*0.74* *(0.57-0.96)*	*1.01* *(0.73-1.38)*	*1.79* *(0.71-4.51)*	*0.97* *(0.69-1.37)*	*0.86* *(0.60-1.23)*	*1.47* *(0.99-2.16)*	*1.03* *(0.77-1.4)*	*1.48* *(0.83-2.64)*	*1.31* *(0.59-2.88)*	*0.66* *(0.41-1.05)*	*0.92* *(0.83-1.02)*	*Q = 18.51* *I^2 ^= 46% *	*0* *(0-23)*
***PAPF***	*0*	*0*	*0*	*21%*	*0*	*0*	*20%*	*2%*	*23%*	*15%*	*0*		*(0-73)*	
**TOTAL PAPF**	62.4%	48.4%	63.8%	63.8%	40.1%	45.0%	58.0%	54.0%	74.2%	61.2%	47.2%			

¶Adjusted for age, sex, education and marital status; NC = not calculated because of small sample size; † Chronic obstructive pulmonary disease; ‡ Myocardial infarction; Rows in Italic show conditions not statistically associated with dependence which have positive PAPF values

Direct standardization for age, sex and education had little effect on the variation in the prevalence of dependence between sites (Table 4). Variation was substantially reduced after standardizing additionally for compositional differences in the main chronic disease determinants of dependence, after which prevalence appeared to be lower in rural sites in Latin America and China and in rural and urban India.

**Table 4:** The prevalence of dependence, before and after direct standardization for demographic and health correlates

	*Crude Prevalence (95% CI)*	*Standardized Prevalence (95% CI)†*	*Standardized Prevalence (95% CI) ‡*
**Cuba**	10.0% (8.9-11.2)	10.5% (8.6-12.4)	4.5% (3.6-5.4)
**Dominican Republic**	11.8% (10.4-13.2)	9.7% (8.4-11.0)	4.1% (3.6-4.5)
**Urban Peru**	9.7% (8.1-11.4)	8.0% (6.2-9.8)	3.1% (2.6-3.5)
**Rural Peru**	4.7% (2.9-6.5)	5.2% (3.2-7.2)	2.0% (1.4-2.6)
**Venezuela**	10.6% (9.2-12.0)	10.9% (9.2-12.6)	4.5% (4.0-4.9)
**Urban Mexico**	11.3% (9.2-13.5)	10.3% (8.4-12.1)	4.5% (3.8-5.2)
**Rural Mexico**	8.2% (6.4-9.9)	7.6% (5.6-9.7)	3.0% (2.4-3.5)
**Urban China**	15.7% (13.6-17.9)	16.0% (13.7-18.2)	5.3% (4.7-5.9)
**Rural China**	5.4% (3.9-6.8)	4.0% (2.8-5.3)	1.1% (0.8-1.4)
**Urban India**	2.9% (1.8-4.01)	3.9% (2.2-5.7)	1.0% (0.6-1.3)
**Rural India**	8.5% (6.7-10.2)	3.7% (2.9-4.5)	2.2% (1.3-3.2)

† Direct standardization for age, sex and education

‡ Direct standardization for age, sex, education, dementia, limb weakness, stroke, depression, eyesight problems and arthritis/rheumatism

## Discussion

Findings from this study show that the prevalence of dependence increased sharply with increasing age, was higher among women than men, and among those with least education. Overall prevalence for those aged 65 years and over varied among 10/66 sites, from 2.9% in urban India to 15.7% in urban China. In most other sites, the indirectly standardized prevalence of dependence was one half to three quarters of that in the USA National Long Term Care Survey. The tendency towards a lower prevalence in rural sites in Latin America and China and in rural and urban India was accentuated after directly standardizing for demographic and chronic disease status. Dementia emerged as by far the leading independent chronic disease contributor to dependence. Limb weakness, stroke, depression, eyesight problems and arthritis made more modest contributions.

The main strengths of our study are the standardised design and assessment procedures carried out in representative catchment area samples across seven MIC, providing reliable and harmonized data on a wide range of cognitive, mental and physical morbidity among older people. This facilitates international cross-cultural comparisons regarding the prevalence and correlates of dependence. The main weakness was that dependence was ascertained using a semi-structured interview, and the rating of level of dependence was somewhat subjective. We chose this pragmatic approach, in the absence of previous research in LMIC, given the difficulties of developing a more structured assessment with demonstrable validity across many different countries and cultures. Other studies have inferred dependence from limitations in core activities of daily living, usually ascertained from the participant. Our approach was more direct, and the ascertainment of needs for care from the care provider, rather than the care recipient may have avoided under-reporting due to social desirability or cognitive impairment. Data on the inter-rater reliability of our assessment would have been valuable. Future cross-cultural comparisons would be assisted by a clearer operational definition of the construct. We did not cover the effects of cancer, endocrine disorders, genitor-urinary conditions and oral conditions on dependence, but these were likely to have been minor [31]. More importantly, different conditions were ascertained with different levels of rigour; dementia, depression and hypertension by clinical assessment, but heart disease and stroke by self-report of medical diagnosis and visual and hearing impairment by self-reported impairment. Assuming random misclassification, this may have tended to reduce the size of any observed effect on dependence towards the null. The problem of self-report has been discussed by Amartya Sen who proposed that 'people in states that provide more education and better health facilities are in a better position to diagnose and perceive their own morbidities than are the people in less advantaged states, where there is less awareness of treatable conditions (to be distinguished from "natural" states of being)' [32]. Finally, our data are cross-sectional. Therefore we cannot infer causality from the observed associations between health conditions and dependence. Some associations might have been inflated by reverse causality, thus depression can be a consequence as well as a cause of dependence [5,33]. Information bias may also have occurred, since interviewers' ratings of the informant's account of needs for care may have been influenced by knowledge of the participant's health status.

Our estimates of the crude prevalence of dependence among those aged 65 years and over in MIC are generally lower than those reported in previous population-based studies of older people in high income countries; in England and Wales [34] (15.7% with significant disability among whom 86% had dependency needs), Scotland [35] (15% with short interval dependence), Spain [36] (15.5% with dependence in one or more of seven ADLs), France [37] (12.4% confined to home or bed) and the USA National Long Term Care Survey [29] (17.1% disabled in one or more activities of daily living, or living in a care home). Indirect standardisation, using the age-specific prevalences reported in the last of these studies confirmed this impression for all sites other than urban China. A relatively lower age-specific prevalence of dependence in MIC may be explained by a lower prevalence of chronic disease. Alternatively, given that prevalence is the product of incidence and duration, it may be that survival in a state of dependence is much shorter in MIC settings. We found, after standardizing for the main chronic disease correlates of dependence, that prevalence was lower in rural sites in Latin America and China and in rural and urban sites in India. This suggests another possible explanation. In these traditional and less developed settings, where most older people live with their children and are routinely provided with support for both core and instrumental activities of daily living, it may be difficult to identify 'the need for frequent human help or care beyond that habitually required by a healthy adult'. In Egypt, urbanisation has contributed to a growing awareness of unmet needs for care among older people; poor immigrant families living in slum districts need to work to maximise household income, leaving dependent older relatives without assistance [38].

We found, consistently across a wide range of MIC settings, that dementia is by far the largest contributor to dependence in the older population. This finding is analogous to that on the correlates of disability from the same 10/66 group surveys [39] although the effect sizes and population attributable prevalence fractions for the association with dementia are much larger for dependence than for disability. Other neurological and neuropsychiatric conditions - limb paralysis or weakness, stroke and depression - featured prominently in the list of leading contributors to dependence. This pattern of findings is entirely consistent with a large body of literature from high income countries. In a cohort study of Medicare recipients in the USA the onset of dementia at 12 months was strongly associated with the onset of dependence by 36 months (adjusted OR 7.5), low body mass index (OR 6.1), psychiatric disorder (OR 4.5), stroke (OR 2.5) and obesity (OR 2.1) also being independently associated. The onset of coronary heart disease, cancer, hypertension, lung disease, diabetes and hip fracture did not predict dependence [40]. Similar findings were reported from a three year follow-up of a population-based cohort study in Sweden [41]. Predictors of institutionalisation were very similar in a meta-analysis of 77 longitudinal community-based studies from the USA [42]. Cognitive impairment was the strongest predictor of institutionalisation (RR 2.54), the increased risks associated with cancer (RR 1.15), hypertension (RR 1.04) and diabetes (RR 1.35) being modest in comparison; there were no associations observed with cardiovascular disease, arthritis, or lung disease. In Sweden, the population attributable fraction for the association between dementia and incident institutionalisation was 61% [43].

The gradient in the prevalence of dependence among older people, between HIC and MIC, and between urban and rural and least and more developed sites in our surveys suggests the potential for a substantial shift in the global profile of dependence, occurring mainly in low and middle income countries, and linked both to rapid demographic ageing and the health transition. There will be unprecedentedly rapid increases in the numbers of older people, and the prevalence of chronic diseases amongst them. Dependence, a consequence of chronic disease disability, will increasingly come to dominate the health and social care agendas in these countries. The proportions of dependent persons who are aged 60 and over will increase between 2000 and 2050, from 21% to 30% in sub-Saharan Africa, from 23% to 44% in India, from 23% to 47% in Latin America, from 30% to 60% in China, compared with from 45% to 61% in HIC [44]. Over this period numbers of dependent older people are forecast to quadruple in most LMIC, while numbers of dependent younger people remain relatively stable. Therefore, in all world regions dependence is rapidly becoming a problem associated with ageing processes, particularly chronic disease morbidity. In the USA, compression of morbidity [45] was observed to have occurred in successive cohorts enrolled into the American's Changing Lives (ACL) study [46]. Thus, at least for those with higher levels of education, increases in life expectancy comprised additional years of healthy life, rather than years lived with disability. For the least educated the pattern of a linear decline in health and functional status persisted in successive cohorts. As the demographic and health transitions impact on LMIC, the extent to which the chronic disease epidemics are prevented and controlled, and the extent to which improvements in public health and clinical care are equitably distributed will have a major impact on future long-term care requirements, and the attendant societal costs. There is an urgent need for these trends to be monitored in LMIC, using similar methodologies to the ACL studies.

Preventive interventions targeting older dependent people should be prioritised, mindful that according to the compression of morbidity hypothesis, healthy ageing, and healthy lifestyles may postpone the onset of chronic ill health and disability in the final years of life. Regardless of the success of such initiatives, numbers of dependent older people will increase markedly in the coming decades particularly in MIC, and the dependency ratio (the ratio of the dependent population to the 'working-age' population) is also set to increase from 8% to 14% in China and from 9% to 12% in India, compared with from 7% to 10% in developed countries [5]. Under the most pessimistic scenario, by 2050 the dependency ratio will have reached 20% in China. It is therefore imperative that LMIC make policies and plans for the future provision and financing of long-term care [47]. Some expansion of the care home sector from a very low base seems inevitable, regardless of government and cultural disapproval [17]. This process needs to be monitored, and the emerging industry needs to be regulated for quality of care. As a counterpoint, informal care can be incentivised through the provision of non-means tested pensions for older people, and compensatory disability and caregiver benefits [4,48]. Most importantly, family caregivers need to be supported in their role, a task currently neglected by community healthcare services [49]. Initial findings from randomised controlled trials of our 'Helping Carers to Care' intervention, for caregivers of people with dementia in Moscow [50] and India [51] suggest considerable potential benefits for caregiver strain. Implementation of such interventions and policies will be a challenge in resource-poor LMIC. The World Health Organisation through its Mental Health Gap Action Programme (mhGAP) is finalising the development of evidence-based guidelines for the treatment of mental and neurological disorders by non-specialist health care workers in LMIC, providing for the first time a feasible and cost-effective model for scaling up services in these regions [52]. Dementia and depression are two of the eight priority disorders, and the teams involved in developing the guidelines have recently published outlines of packages of care for these conditions [53,54].

## Conclusion

To our knowledge this is the first cross cultural study to investigate the contribution of chronic diseases to dependence in a population from middle income countries. Our results showed that old age, female sex and lower education were associated to dependence defined as the need of help to perform activities of daily living beyond that habitually required by healthy individuals. Among chronic conditions, dementia was by far the largest contributor to dependence among this population. Other substantial contributors were limb impairment, stroke, and depression. Our results suggest that with the estimated increase of the elderly population and dementia, dependence is likely to become an important health issue. Preventive interventions targeting older dependent people should be prioritised.

## List of Abbreviations used

LMIC: low and middle income countries; PAPF: population attributable prevalence fraction; 10/66 DRG: 10/66 Dementia Research Group; WHO: World Health Organization; GBD: Global Burden of Disease; SMR: standardized morbidity ratios; USA: United States of America; GMS: Geriatric Mental State; ICD: International Classification of Diseases; DSM: Diagnostic and Statistical Manual of Mental Disorders; COPD: Chronic Obstructive Coronary Disease; OR: odds ratio; PR: prevalence ratio; ACL: American's Changing Lives study; mhGAP: Mental Health Gap Action Programme

## Competing interests

The authors declare that they have no competing interests.

## Authors' contributions

RMS carried out the analyses and wrote the first draft with assistance and revision from MP. MP leads the 10/66 Dementia Research group and CPF acts as research coordinator. JJLR (Cuba), DA (Dominican Republic), MG (Peru), AS (Venezuela), ALS (Mexico), KSJ (Vellore, India), JW (Chennai, India), and YH (China) were principal investigators responsible for the fieldwork in their respective countries. ATJ (Chennai, India), MAGH (Marianao, Cuba), GRP (Dominican Republic), ZL (China), and TZ (Mexico) were research coordinators on their local site and worked closely to the local principal investigator in the data collection. All authors reviewed the manuscript and provided further contributions and suggestions. All authors read and approved the final version of the manuscript.

## Pre-publication history

The pre-publication history for this paper can be accessed here:

http://www.biomedcentral.com/1471-2318/10/53/prepub
